# Optical characterization of inhomogeneity of polymer-like thin films arising in the initial phase of plasma-enhanced chemical vapor deposition

**DOI:** 10.1016/j.heliyon.2024.e27246

**Published:** 2024-03-01

**Authors:** Jan Dvořák, Jiří Vohánka, Vilma Buršíková, Ivan Ohlídal

**Affiliations:** Department of Plasma Physics and Technology, Faculty of Science, Masaryk University, Kotlářská 2, Brno, 611 37, Czech Republic

**Keywords:** Ellipsometry, Reflectometry, Optical characterization, Polymer-like thin films, Plasma polymer, Inhomogeneity

## Abstract

In this study, an optical investigation in a wide spectral range of polymer-like (SiO_*x*_C_*y*_H_*z*_) thin films deposited by plasma-enhanced chemical vapor deposition (PECVD) is presented. The primary focus is on assessing the homogeneity of the grown films. Within the PECVD, it is possible to alter the properties of the deposited material by continually adjusting deposition process parameters and hence allow for the growth of inhomogeneous layers. However, as shown in this study, the growth of homogeneous layers could be similarly challenging. This challenge is especially pronounced at the beginning of the deposition process, where it is necessary to consider the influence of the substrate among other factors, as even slight variations in the deposition conditions can lead to the formation of inhomogeneous layers. Several series of polymer-like thin films were deposited onto silicon substrates with the goal of producing homogeneous layers, i.e. all deposition parameters were held constant. These samples were optically characterized with a special interest in homogeneity, especially at the beginning of the growth. It was found that initial inhomogeneous growth is always present. The thickness of the initial inhomogeneous part was found to be surprisingly large.

## 1 Introduction

Polymer-like materials (also known as plasma polymers) are a wide family of materials with a broad spectrum of optical and mechanical properties, which can be described by a variety of chemical formulae. In general, the term plasma polymer denotes a material created as a result of the passage of an organic vapor through the glow discharge. In spite of the use of the word polymer, polymer-like thin films have only a little in common with conventional polymers [1]. Particularly, they display a more random molecular arrangement, deviating from the well-defined chemical structure of the monomers employed in their synthesis [2]. This distinction arises due to the unique processing conditions involving plasma-based reactions during plasma-enhanced chemical vapor deposition (PECVD), leading to the formation of amorphous structures with diverse bonding configurations. These variations in molecular architecture grant polymer-like materials intriguing properties, rendering them attractive for a wide array of applications in nanotechnology, surface engineering, biocompatible coatings [3], transparent barrier films in packing technology [4], or anti-corrosion coatings [5].

In this work, we focus on the plasma polymers satisfying the composition SiO_*x*_C_*y*_H_*z*_ [6], [7]. These materials find primary application as protective coatings for substrates made of plastics, such as polycarbonate [8], [9]. During the deposition process, the properties of the resulting films can be systematically modified by adjusting the deposition conditions. This allows for a continuous transition from a softer, transparent material with excellent substrate adhesion to a harder, albeit more absorptive material. Through this controlled process, it becomes possible to grow the protective coatings that exhibit very good adhesion to the substrate [10], [11], [12].

While these films are predominantly employed as protective coatings with thicknesses in the order of micrometers, the use of these layers as optical films could also be of high interest. Several authors have directed their investigations towards the mechanical and structural characteristics of these films [13], [14]. However, the optical properties have been somewhat neglected. Nevertheless, some studies have focused on the optical properties of such protective films. Due to the high absorption in the top protective part of the films, obtained information was restricted primarily to the uppermost region of the sample [15]. Moreover, optical studies usually focus on limited spectral regions [16], especially the infrared [17]. For many practical applications, this methodology is entirely adequate. Others employed more thorough investigations of inhomogeneous layers [18], [19], but within these investigations, it is impossible to distinguish inhomogeneity caused by the varying deposition parameters and the inherent initial inhomogeneity which is always present at the beginning of the deposition. Most of the time the latter can be neglected due to the small thickness and therefore small influence on the resulting film, but when the initial inherent inhomogeneity has a non-negligible thickness, this is no longer possible.

Note that the optical characterization of inhomogeneous thin films is more complicated than that of homogeneous thin films for two main reasons. Firstly, inhomogeneous thin films involve a larger number of parameters that need to be determined. Secondly, the characterization of inhomogeneous thin films requires the use of more complicated mathematical formulae, procedures, and structural models when compared to homogeneous thin films.

The foregoing statements are illustrated, for example, in characterizing homogeneous thin films in papers [20], [21], [22], [23], [24], [25] and inhomogeneous thin films in papers [26], [27], [28], [29], [30], [31]. It should be emphasized that a correct choice of both the structural and dispersion models represents a necessary condition for the successful and correct optical characterization of inhomogeneous thin films. The optical characterization of homogeneous and inhomogeneous thin films is especially difficult if these films exhibit defects such as transition layers, overlayers, thickness non-uniformity, random roughness of their boundaries, etc. A sufficient attention has not been devoted to the optical characterization of the thin films with these defects. Despite this reality, a certain number of papers dealing with the methods for the optical characterization of the films exhibiting the defects mentioned above has been published so far. For example, the films with the transition layers and overlayers have been characterized in papers [32] and [33], respectively, while the films exhibiting thickness non-uniformity and boundary roughness have been characterized in papers [34], [35], [36], [37] and [38], [39], [40], [41], respectively. In this work, the defect consisting in transition layers placed between the silicon substrates and characterized polymer-like thin films will be taken into account.

In this work, it was found that the studied polymer-like thin films exhibited inhomogeneity, even though our intention was to prepare homogeneous films. In the optical characterization of these films, this inhomogeneity was manifested by the change in the optical constants and it was largest in the regions adjacent to the substrate, i.e. in the parts of films deposited in the initial phase of their growth. It is important to take this inhomogeneity into account if these films are studied by optical methods, otherwise the obtained results can be inaccurate or misrepresented. In many applications it is necessary prepare coatings consisting of polymer-like films tailored for specific optical properties [42], such as antireflection coatings, optical filters, etc. These coatings utilize stacks of films with different refractive indices or inhomogeneous films with suitably chosen refractive index profiles [42]. It is evident, that the mentioned inhomogeneity must be taken into account in the design of these coatings.

## 2 Experiment

In our study, we conducted optical characterization on four series of samples of polymer-like thin films deposited onto silicon single-crystal substrates. The difference between the individual series was the flow rate of working gas in the deposition process, and hence different optical and structural properties of the films. Each series comprised four samples, which differed in deposition time and, consequently, in the thickness of the deposited film. Samples within each series were processed simultaneously using a multi-sample method [43]. It was assumed that thin films exhibit initial inhomogeneity which diminishes after reaching a certain thickness, and the rest of the films grow homogeneously. The profiles of the inhomogeneous parts of the films were assumed to be the same throughout the samples within each series. In other words, all dispersion parameters as well as the structural parameters were assumed to be the same for all samples within the series, except for the thickness of the films. This approach leads to the reliable description of initial inhomogeneity with respect to the deposition conditions.

### 2.1 Sample preparation

All samples within each series were deposited in exactly the same way, with an abrupt stop of deposition at specific times (120 s, 240 s, 360 s, and 480 s).

The films under study were deposited onto silicon single-crystal substrates using PECVD in a capacitively coupled radiofrequency-glow discharge configuration at a frequency of 13.56 MHz. This setup consisted of a glass cylinder sealed by two stainless steel flanges, housing two parallel graphite electrodes for the deposition process. The lower electrode received the RF power while maintaining a negative DC self-bias voltage to regulate ion bombardment on the sample surface. Before the film deposition, the substrates underwent a 5-minute pretreatment in an argon discharge under specific conditions: 50 W of applied power, a bias voltage of -240 V, and an argon flow rate of 5 sccm. The deposition discharge took place within a methane (CH_4_) and hexamethyldisiloxane (C_6_H_18_Si_2_O - HMDSO) mixture, introduced into the reactor chamber through a glass torus equipped with multiple outlets to ensure uniform gas distribution. The HMDSO flow rate was maintained at 1 sccm throughout the process.

Each series is distinguished by the methane (CH_4_) flow rate, specifically 0, 2.5, 5, and 7.5 sccm, which consequently influenced the deposition pressure. These series will be further denoted as series #1, #2, #3 and #4, respectively. Deposition conditions were held constant during the deposition, for 8, 6, 4, and 2 minutes to gain the samples with different thicknesses. Individual samples were numbered from thickest to thinnest, i.e. an 8-minute deposition is designated as Sample #1, a 6-minute deposition as Sample #2, and so on. The supplied power was 50 W.

### 2.2 Experimental arrangement

Experimental data were collected using the Horiba Jobin Yvon UVISEL variable angle spectroscopic phase-modulated ellipsometer and the Perkin Elmer Lambda 1050 spectrophotometer. Detailed descriptions of the experimental setups for these instruments can be found, for example, in the references [44], [45].

The reflectance was obtained at near-normal incidence 6^∘^, the transmittance was obtained at normal incidence, while the ellipsometric data were acquired at angles of incidence ranging from 55^∘^ to 75^∘^ in 5^∘^ increments. The spectral range covered by the data was 0.6–6.5 eV (190–2067 nm) and 190–1800 nm (0.69–6.5 eV) for ellipsometric and spectrophotometric measurements, respectively.

The experimental data for each sample comprises ellipsometric and reflectometric data from the film side, the reflectance from the back side, and the transmittance. However, the reflectance from the back side and transmittance were measured only in the NIR region (865–1800 nm) where the silicon substrate is transparent.

In the case of phase-modulated ellipsometers, the measured quantities are usually not represented by classical ellipsometric angles (Ψ, Δ) but rather by so-called associated ellipsometric parameters $I_{\text{s}}$, $I_{\text{c}}$, $I_{\text{n}}$, which are related to Ψ and Δ as:

$$I_{s}=P\sin2\Psi \sin\Delta ,I_{c}=P\sin2\Psi \cos\Delta ,I_{n}=P\cos2\Psi ,$$

where *P* represents the degree of polarization. These parameters correspond to the three independent elements of the normalized Mueller matrix of isotropic systems, which can be expressed as:

$$M=R\left(\begin{array}{cccc} 1 & -I_{n} & 0 & 0\\ -I_{n} & 1 & 0 & 0\\ 0 & 0 & I_{c} & I_{s}\\ 0 & 0 & -I_{s} & I_{c} \end{array}\right),$$

where $R=(R_{\text{p}}+R_{\text{s}})/2$ is the average of reflectances for p- and s-polarized light. When combined, $I_{\text{s}}$ and $I_{\text{c}}$ provide an accurate measurement of Δ over the full range from $0^{\circ }$ to $360^{\circ }$ and $I_{\text{s}}$ and $I_{\text{n}}$ provide an accurate measurement of Ψ over the full range from $0^{\circ }$ to $90^{\circ }$.

## 3 Structural model

The following assumptions were used to construct the structural model of the samples. The base of the sample is the double-side polished silicon substrate on which the polymer-like thin film is deposited. Between the substrate and polymer-like thin film, there is a transition layer, which corresponds mostly to the thin region of the substrate perturbed by the pretreatment in argon discharge. In the structural model, it is represented by a thin homogeneous layer.

The polymer-like thin film itself is assumed to be inhomogeneous with the same profile in optical constants for all samples within the given series.

**Figure 1:**
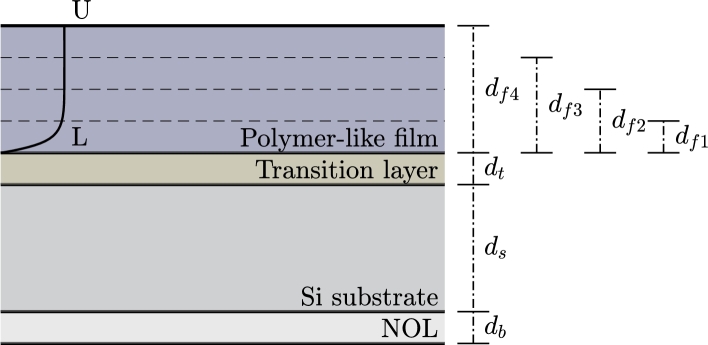
Schematic illustration of the structural model of the samples. Dashed lines illustrate the top surface of the samples with different thicknesses.

All boundaries are perfectly smooth and parallel. A very thin native oxide layer (NOL) was assumed on the back side of the silicon substrate. Optical constants of this layer were assumed to be the same as those of SiO_2_. While in reality, the NOL might exhibit slightly different optical properties, the subtle difference from properties of SiO_2_ has little influence on the final results. The situation is schematically illustrated in Fig. 1.

## 4 Dispersion model

### 4.1 Dispersion model of polymer-like thin film

The dielectric response of the polymer-like thin film is modeled based on the universal dispersion model (UDM) [46], [47], which allows for the construction of complex models that satisfy fundamental physical requirements, including the time-reversal symmetry, the Kramers–Kronig relations, and consistency with the sum rules. The dielectric response in this model is represented as a sum of contributions, which allow modeling of various absorption processes.

Polymer-like thin films that contain higher proportions of carbon, especially those transitioning to diamond-like carbon material, often exhibit intricate dielectric responses. To accurately interpret their dielectric response, it is essential to consider the sp^2^ and sp^3^ orbital hybridization [1], [42], [48]. Within the dielectric response model, it is beneficial to differentiate between contributions from *σ* electrons, which are associated with stronger bonding, and contributions from weakly bonded *π* electrons.

To account for these distinctions, our model describes the interband transitions using two separate (partially overlapping) absorption bands, each characterized by its own band-gap energy. Specifically, the susceptibility is formulated as follows:

$$\overset{ˆ}{\chi }(E)=N_{0,\sigma }\;{\overset{ˆ}{\chi }}_{\mathrm{ibt}}^{0}(E;E_{g,\sigma })+N_{\sigma }\;{\overset{ˆ}{\chi }}_{\mathrm{CC}}^{0}(E;E_{g,\sigma },E_{c,\sigma },B_{\sigma })+N_{\pi }\;{\overset{ˆ}{\chi }}_{\mathrm{CC}}^{0}(E;E_{g,\pi },E_{c,\pi },B_{\pi }),$$

where the first two contributions pertain to the absorption band associated with *σ* electrons exhibiting stronger bonding, while the final contribution relates to the absorption band corresponding to weakly bonded *π* electrons. The functions denoted with superscript 0 represent susceptibility contributions that have been normalized using the sum rule integral:

$$\int_{0}^{\infty }E\chi_{\nu ,i}^{0}(E;\ldots )dE=1,$$

where the symbols ν=ibt,CC distinguishes specific normalized contributions to susceptibility, while $\chi_{\nu ,i}^{0}(E;\ldots )$ denotes their imaginary parts. The strengths of individual contributions are determined by the factors $N_{\sigma ,0}$, $N_{\sigma }$, and $N_{\pi }$. The quantities $E_{g,\sigma }$ and $E_{g,\pi }$ correspond to the band gap energies of the two distinct absorption bands while parameters $E_{c,j}$ (where $E_{c,j}>E_{g,j}$) and $B_{j}$ are responsible for determining the position and width of the absorption peak. The total transition strength, which can be evaluated using the sum rule integral, is explicitly given by:

$$\int_{0}^{\infty }E\chi_{i}(E)dE=N_{0,\sigma }+N_{\sigma }+N_{\pi },$$

where $\chi_{i}(E)$ is the imaginary part of susceptibility. The contribution ${\overset{ˆ}{\chi }}_{\mathrm{ibt}}^{0}(E;E_{g,\sigma })$ represents the absorption band starting at the band gap energy $E_{g,\sigma }$ and extending to infinity. The contributions ${\overset{ˆ}{\chi }}_{\mathrm{CC}}^{0}(E;E_{g,j},E_{c,j},B_{j})$, with j=σ,π correspond to the Campi–Coriasso dispersion model [47], [49].

To compute the real parts of these contributions, the Kramers–Kronig relations are employed. Further information about the construction of UDM can be found in the reference [50]. The dispersion model describing the response of the polymer-like thin films outlined above was also used in our previous work [51], where the concrete forms of all contributions can be found.

### 4.2 Description of inhomogeneity

The profile of the optical constants is constructed by assuming the profile of the individual dispersion parameters which are in the following form:

(1) $$p_{\alpha }(z)=p_{\alpha }^{\text{U}}+\left(p_{\alpha }^{\text{L}}-p_{\alpha }^{\text{U}}\right){\left(\frac{z+d_{\text{max}}-d}{d_{\text{max}}}\right)}^{s},$$

where $p_{\alpha }$ denotes the particular dispersion parameter, the superscripts *U* and *L* distinguishes the upper and lower boundaries, $d_{\text{max}}$ is the thickness of the thickest sample of the series, *d* is the thickness of the individual film and *z* is the distance from the film upper boundary. Parameter *s* models the shape of the profile, while s=1 results in a linear profile of a particular parameter, the higher values of *s* lead to the stronger inhomogeneity near the film-substrate boundary.

### 4.3 Dispersion model of transition layer

Transition layers have to be taken into account at the boundary between silicon substrates and polymer-like thin films. The emergence of these transition layers can be attributed to the pre-treatment of silicon substrates through argon discharge prior to film deposition, as elaborated in prior work [52]. It is assumed that these thin transition layers are homogeneous and absorbing and are described using the Campi–Coriasso dispersion model with four terms.

Due to the high absorption coefficient in a substantial part of the measured spectral range of the top part of the polymer-like thin film, there is just too little information obtainable about the transition layer. As a result, the dispersion parameters of the transition layer, when fitted together with structural and dispersion parameters of polymer-like thin film, tend to unrealistic values. Therefore, the optical constants of the transition layer were obtained separately by optical characterization of the substrate pretreated in argon discharge but without deposited layer. During this separate characterization, the thin NOL layer was considered as an overlayer originating from air exposure, which would not have occurred if the polymer-like thin film had been deposited immediately after cleaning procedure. The reliability of the determined optical constants of the transition layer is supported by the strong agreement with our earlier work [35], [53], [54]. The only parameter related to the transition layer determined during the processing of data for samples with films was its thickness. Moreover, the same thickness was used for all samples in all series.

## 5 Data processing

To enhance the robustness of the fits, the multi-sample method was utilized using newAD2 software developed by our research group [55]. This method enables to use of some of the structural and dispersion parameters across the samples of each series. As mentioned before, all the samples within each series were assumed to have the same dispersion parameters of the bottom part of the polymer-like thin film and the same profile of dispersion parameters.

### 5.1 Methods employed for expressing the optical quantities of inhomogeneous films

The optical quantities of the inhomogeneous layer were calculated using the multilayer approximation method, which describes the polymer-like thin film as a stack of homogeneous sublayers approximating its inhomogeneous profile. The approximation with 65 sublayers was used, which was sufficient to calculate the optical quantities with accuracy much better than their measurement errors.

An alternative approach based on the multiple-beam interference method described in [56] was also tried. In this approach, the reflection or transmission coefficient is given as a sum of contributions representing the Wentzel–Kramers–Brillouin–Jeffreys (WKBJ) approximation and corrections representing internal reflections inside the inhomogeneous layer. The WKBJ approximation by itself was not sufficient and it was necessary to include corrections for one and two internal reflections inside the inhomogeneous layers to reach the sufficient accuracy of calculations.

The results presented in the tables and figures were achieved using the multilayer approximation method, however, apart from small numerical errors the multiple-beam interference method gives the same results.

## 6 Results and discussion

### 6.1 Measurement results

In this section, the results of optical characterization are presented. As previously stated, the entire set of samples consists of four series, each containing four samples, totaling 16 samples. Due to limited space, the presentation of measured data will be confined to two samples: the thinnest and thickest from series #4. However, it should be noted that the quality of the fits is comparable across the entire sample set.

**Figure 2:**
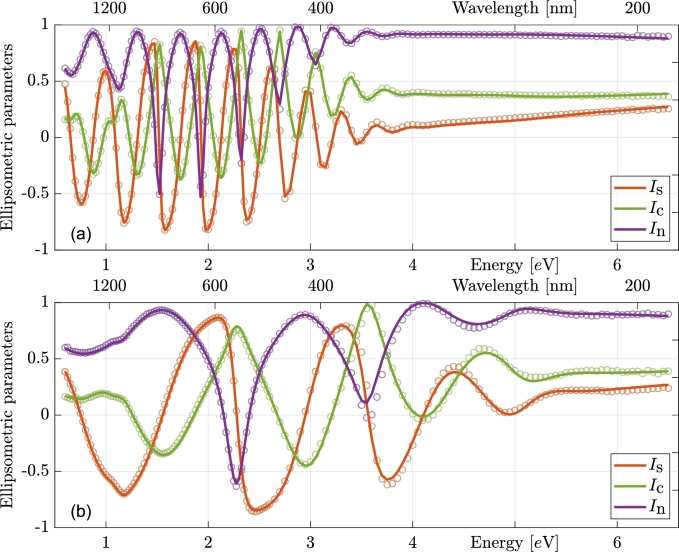
Measured ellipsometric parameters with their respective fits. AOI = 70^∘^. The top panel (a) represents the thickest sample, and the bottom (b) is the thinnest sample from series #4. Points represent measured data, lines fitted curves.

Ellipsometric measurements of the mentioned samples and their corresponding fits are illustrated in Fig. 2. It is evident that there is excellent agreement between the measurements and the theoretical curves. Additionally, in the figure, a distinct difference in the density of measured points is observed below approximately 2.5 eV compared to above. This discrepancy arises from the UV sensitivity of the studied films, necessitating the division of each measurement into two parts. The used ellipsometer follows the sequence Source–Polarizer–Sample–Compensator–Analyzer–Monochromator–Detector, which is suboptimal for UV-sensitive samples, as it exposes the sample to the high-intensity light beam throughout the entire measurement process. To mitigate this, the NIR-visible part of the spectrum is measured with the UV filter placed after the light source, while the UV part is measured without filter but using notably faster procedure with lower density of measured points.

**Figure 3:**
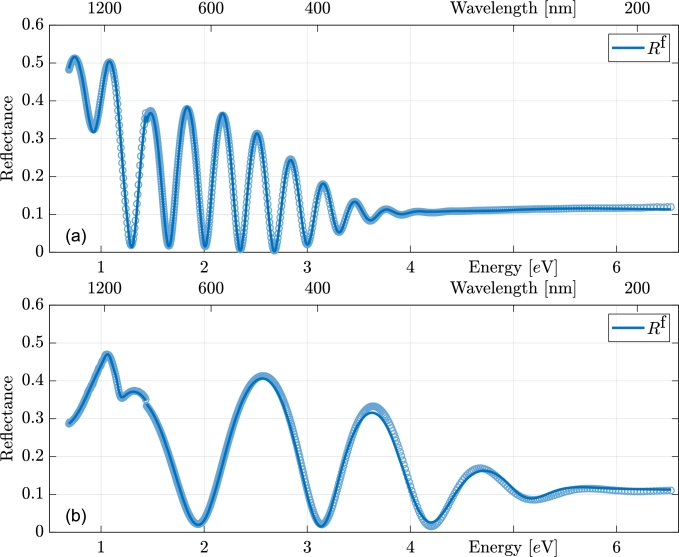
Measured reflectance data from the film side with their respective fits. The top panel (a) represents the thickest sample and the bottom (b) thinnest from series #4. Points represent measured data, lines fitted curves.

**Figure 4:**
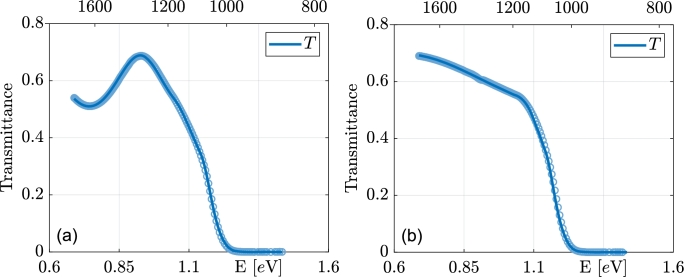
Measured transmittance data in the NIR region with their respective fits. The left panel (a) represents the thickest sample and the right (b) thinnest from series #4. Points represent measured data, lines fitted curves.

Fig. 3 illustrates the reflectance measurements along with their corresponding fits, demonstrating excellent agreement. The small mismatch in data around 1.5 eV is because the NIR and visible-UV reflectances were measured separately. Nevertheless, our fitting procedure accounts for this mismatch by means of corrections for the systematic errors of the instrument. The transmittance data are shown in the Fig. 4.

### 6.2 Optical constants

**Figure 5:**
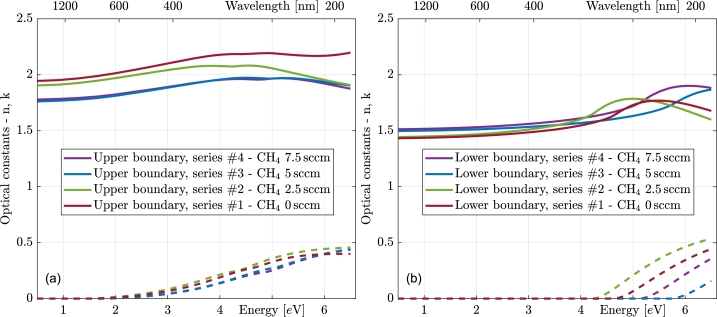
Optical constants (solid lines refractive index, dashed extinction coefficient) of the thin films at the upper (left panel (a)) and lower (right panel (b)) boundaries of the thickest films in individual series.

The optical constants and dispersion parameters of the polymer-like thin films are depicted and summarized in Fig. 5, and Table 1, Table 2. When focusing on the optical constants near the top of the grown film, it is verified that lower CH_4_ content leads to a higher refractive index of the grown layer [57], [58], [59]. However, no clear trend can be seen near the bottom boundaries. In general, it could be said that higher CH_4_ content leads to a higher refractive index and a higher band gap energy in this part of the growth, but the trend is not that strong, and clearly, some other factors influence the growth at this stage.

**Table 1:** Dispersion parameters of the top boundaries of the thickest films. Stars indicate parameters whose values were fixed in said values.

Parameter			Series #1 Top	Series #2 Top	Series #3 Top	Series #4 Top
Transition strength of *σ* electrons – ibt term	*N*_0,*σ*_	[eV^2^]	468	620	491	638
Band gap energy of *σ* electrons	*E*_g,*σ*_	[eV]	4.56	4.27	4.97	4.81
Transition strength of *σ* electrons – CC term	*N*_*σ*_	[eV^2^]	0.83	1.44	0.43	0.85
Peak position of *σ* electrons	*E*_c,*σ*_	[eV]	9.35	13.37	10.89	14.80
Peak broadening of *σ* electrons	*B*_*σ*_	[eV]	0.5*	0.5*	0.5*	0.5*
Transition strength of *π* electrons	*N*_*π*_	[eV^2^]	26.55	20.1	26.3	20.5
Band gap energy of *π* electrons	*E*_g*π*_	[eV]	1.51	1.36	1.74	1.75
Peak position of *π* electrons	*E*_c*π*_	[eV]	5.05	4.69	5.41	5.19
Peak broadening of *π* electrons	*B*_*π*_	[eV]	2.77	2.67	2.88	2.75

**Table 2:** Dispersion parameters of the bottom boundaries of the polymer-like thin films. Stars indicate parameters whose values were fixed in said values. The dagger sign indicates that the parameter is tied to the same parameter of the top boundary of the respective series.

Parameter			Series #1 Bottom	Series #2 Bottom	Series #3 Bottom	Series #4 Bottom
Transition strength of *σ* electrons – ibt term	*N*_0,*σ*_	[eV^2^]	351	302	635	528
Band gap energy of *σ* electrons	*E*_g,*σ*_	[eV]	4.62	4.22	5.71	5.11
Transition strength of *σ* electrons – CC term	*N*_*σ*_	[eV^2^]	0*	0*	0*	0*
Peak position of *σ* electrons	*E*_c,*σ*_	[eV]	†	†	†	†
Peak broadening of *σ* electrons	*B*_*σ*_	[eV]	0.5*	0.5*	0.5*	0.5*
Transition strength of *π* electrons	*N*_*π*_	[eV^2^]	0*	0*	0*	0*
Band gap energy of *π* electrons	*E*_g*π*_	[eV]	†	†	†	†
Peak position of *π* electrons	*E*_c*π*_	[eV]	†	†	†	†
Peak broadening of *π* electrons	*B*_*π*_	[eV]	†	†	†	†

Table 1, Table 2 show the specific values of the obtained dispersion parameters. The broadening of the *σ* electron peak had to be fixed (indicated by the star superscript) at 0.5 eV. This is due to the position of this peak lying beyond the spectral range of our instrumentation. Consequently, when fitted freely, it tends to unrealistic values. Additionally, during the fitting procedure, the Campi–Coriasso term of sigma electron transition strength ($N_{\sigma }$) and the transition strength of pi electrons ($N_{\pi }$) at the bottom boundary tend toward zero. Consequently, they were fixed at this value, leaving only the interband transition term of sigma electron transition strength at nonzero values at the bottom boundary.

Cross signs denote the parameters whose values are tied to the same parameter at the top of the respective film. This necessity arises from the fact that, due to absorption in a significant part of our spectral range by the upper part of the film, there is too little information obtainable about the lower part of the film. One way to overcome this limitation would be to measure the films deposited onto non-absorbing substrates [51]. This would enable the possibility to perform measurements from both sides of the samples. However, it is important to note that the initial inhomogeneity is strongly influenced by the physical properties of the substrate. Consequently, this approach would lead to measurements on samples that are markedly different from those deposited on silicon.

The influence of substrate material on a deposition process has been demonstrated in several works [60], [61], [62], [63], [64]. Various factors may affect the structure of the growing film, including substrate temperature, resistivity, emissivity, and roughness. In [60], [61], it was experimentally proven and confirmed through MC-PIC computer simulation that the secondary electron yield is also a significant factor influencing film growth, particularly at low pressures. Due to their relatively high energies, electrons incident on the substrate during plasma deposition generate secondary electrons that can significantly impact the plasma density above the substrate. These secondary electrons, originating within the substrate material, play a crucial role in ionization, with the resulting ions pivotal in shaping the structure and properties of the growing film. If the substrate's secondary electron yield differs from that of the film material, the deposition process is primarily governed by the substrate's yield. As the growing film achieves a thickness sufficient to match the penetration depth of the incident electrons, the plasma density above the substrate becomes predominantly influenced by the film's secondary electron yield. This may result in the growth of an inhomogeneous film until a sufficient thickness is reached to suppress the substrate effect.

The electron mean free path depends on the deposition pressure. In the case of relatively low deposition pressures, the dispersion of electrons and ions created by ionization with secondary electrons is low, and their displacement in the radial direction from the substrate is small. Therefore, a larger plasma volume above the substrate is affected by the secondary electron emission yield of the substrate. As the pressure increases, the electron mean free path decreases, and the electrons undergo many collisions with much heavier particles, scattering them more from the position above the substrate. This is evidenced by increased dispersion and the loss of secondary electron energy, leading to a decrease in the penetration depth of incident electrons and a reduction in the film thickness needed to suppress the effect of the secondary electron emission yield of the substrate.

**Figure 6:**
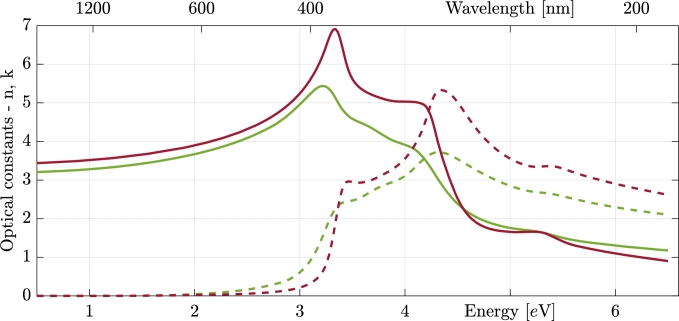
Optical constants (solid lines refractive index, dashed extinction coefficient) of the transition layer (green lines) together with optical constants of a silicon substrate (red lines) for comparison.

Fig. 6 displays the optical constants used for the transition layer, which originates in the plasma cleaning procedure of the substrate. The sample used for this separate characterization of the transition layer was treated in the same way as all the other samples; that is, the substrate was cleaned in the Ar plasma with the same power and for the same amount of time. In the figure, the optical constants of crystalline silicon are also plotted for comparison.

Table 3 summarizes the thicknesses of the polymer-like thin films obtained from optical characterization, along with the thickness of the transition layer. Based on our previous experience, the thickness of the transition layer should be approximately 15 nm [54].

**Table 3:** Resulting thickness of the films and transition layer.

Thickness [nm]	Sample #1	Sample #2	Sample #3	Sample #4
Series #1	842.8	725.5	567.7	382.7
Series #2	790.2	760.3	484.3	379.9
Series #3	1015.7	812.8	525.6	286.4
Series #4	942.8	725.0	507.4	273.4
Transition layer	16.9			

### 6.3 Resulting profiles

In this section, the profiles and thicknesses of initial inhomogeneities will be discussed. Table 4 summarizes the thicknesses of initial inhomogeneities together with several other related parameters. It should be noted that the pressure inside the deposition chamber was measured before and then again after the deposition. Since both values differ only in the order of the experimental error, only one value is shown in the table. As mentioned before, the chosen profile is in the form of Equation (1) and hence has no truly homogeneous part. For the sake of comparison, the thickness of the inhomogeneous part is arbitrarily defined as the thickness between the bottom boundary of the film and the position where the refractive index reaches 95% of the difference between the initial optical constants and optical constants on the top surface. This point is indicated in Fig. 8 by the markers.

**Table 4:** Profile and inhomogeneity parameters. The pressure in the deposition chamber and the flow rate of methane are also shown in the table.

Series	CH_4_ flowrate [sccm]	Deposition pressure [Pa]	Thickness of the inhomogeneous part [nm]	s parameter of the profile
#1	0.0	12	653	1.84
#2	2.5	23	382	4.34
#3	5.0	31	356	5.85
#4	7.5	39	266	8.62

One can observe a trend concerning the deposition conditions, specifically the CH_4_ flow rate, in both the thickness of the inhomogeneous part and the *s* parameter. The *s* parameter is presumed to be the same for all four samples within each series. However, it is worth noting that when fitting the *s* parameter independently for all the samples, the resulting values were very similar. This supports the assumption that the initial inhomogeneity is independent of the final thickness of the samples.

The large values of the inhomogeneity thickness can be somewhat surprising. However, most authors focus on films with thicknesses in the order of micrometers. Thus the ability to investigate the inhomogeneous region is limited, especially if the measurements are performed in a limited spectral range.

**Figure 7:**
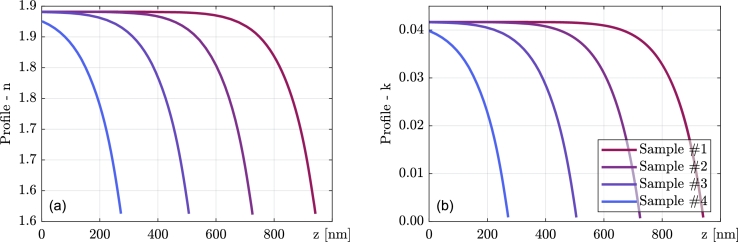
Resulting profile of optical constants (left panel (a) refractive index, right (b) extinction coefficient) for series #4 at *E* = 3 eV. Zero on the x-axis corresponds to the film surface.

**Figure 8:**
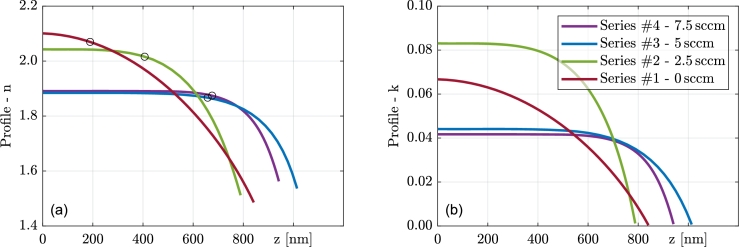
Resulting profile of optical constants (left panel (a) refractive index, right (b) extinction coefficient) for the thickest samples of all the series at *E* = 3 eV. Zero on the x-axis corresponds to the film surface.

In Fig. 7, the profiles of optical constants of series #4 are shown for the chosen photon energy E=3eV. The zero on the x-axis is set to be on the top surface of the sample. One can observe strong inhomogeneity near the beginning of the growth, which stabilizes after a few hundred nanometers. The thickest three samples clearly reached homogeneous growth, but the thinnest one did not. Fig. 8 depicts the same thing but for the thickest samples of each series and clearly shows the influence of CH_4_ flow rate on initial inhomogeneity.

## 7 Conclusion

The family of polymer-like materials consists of materials whose optical and mechanical properties can vary substantially and can be tuned during the PECVD deposition. This fact is widely exploited for the growth of inhomogeneous layers with the desired profile of optical constants and distinct mechanical properties of different parts of the resulting film. During the growth, two causes of inhomogeneity apply at once: deliberate tuning of deposition parameters and unintentional inherent inhomogeneity. These two causes are impossible to distinguish within the growth of the inhomogeneous layer.

In this paper, we focused on the unintentional inherent inhomogeneity and showed that this effect could be much stronger than initially thought and that it is strongly influenced by the deposition conditions. The deposited films exhibited strongest optical inhomogeneity near the substrate, which transitioned into almost homogeneous regions observed at the top parts of the thicker films. In our case, we tuned the flow rate of the working gas (CH_4_), which consequently altered the pressure in the deposition apparatus, and showed that initial inhomogeneity could span from a few hundred nanometers to more than 600 nm. This large thickness could be surprising but is reasonable, as discussed in the paper. The observed inhomogeneity can be explained by the influence of the substrate on the deposition process in the initial phase of the film growth. The different secondary electron yield of the substrate compared with the film is likely one the main contributing factors. This is discussed in detail in section 6.2.

A deeper investigation could be beneficial, not only from optical point of view, but also from structural one, especially thorough XPS study, but this surpasses the scope of this study. There is little information about the initial inhomogeneity of polymer-like thin films in the literature; most of the time, this effect is neglected or immeasurable based on the experiment design. Here, only the influence of the flow rate of the working gas was discussed; however, it is highly probable that other deposition conditions would have a strong influence too. Within this deposition method, it is therefore impossible to grow homogeneous layers; one can only minimize the thickness of the inhomogeneous part of the growth by choosing more suitable deposition conditions, but this effect will never be fully suppressed.

**Funding**

This work was supported by the project LM2023039 funded by 10.13039/501100001823The Ministry of Education, Youth and Sports of the Czech Republic.

## CRediT authorship contribution statement

**Jan Dvořák:** Writing – review & editing, Writing – original draft, Visualization, Methodology, Investigation, Formal analysis, Data curation, Conceptualization. **Jiří Vohánka:** Writing – review & editing, Supervision, Software, Conceptualization. **Vilma Buršíková:** Resources, Methodology, Conceptualization. **Ivan Ohlídal:** Writing – review & editing, Validation, Supervision, Funding acquisition, Conceptualization.

## Declaration of Competing Interest

The authors declare that they have no known competing financial interests or personal relationships that could have appeared to influence the work reported in this paper.

## Data Availability

Data presented in this study are available upon reasonable request from the corresponding author.

## References

[1] Biederman H. (2004).

[2] Nisol B., Reniers F. (2015). Challenges in the characterization of plasma polymers using XPS. J. Electron Spectrosc..

[3] Lou B.S., Wang S.B., Hung S.B., Wang C.J., Lee J.W. (2018). Characterization of plasma polymerized organosilicon thin films deposited on 316L stainless steel. Thin Solid Films.

[4] Lu S.K., Chen S.C., Chen T.H., Lai L.W., Liao R.M., Liu D.S. (2015). Barrier property and mechanical flexibility of stress controlled organosilicon/silicon oxide coatings on plastic substrates. Surf. Coat. Technol..

[5] Deng Q., Li W., Zhu L., Chen H., Ju P., Liu H. (2018). Ultrathin, highly anticorrosive and hydrophobic film for metal protection based on a composite organosilicon structure. Colloids Surf. A, Physicochem. Eng. Asp..

[6] Gosar Ž., Kovač J., Mozetič M., Primc G., Vesel A., Zaplotnik R. (2019). Deposition of SiO_*x*_C_*y*_H_*z*_ protective coatings on polymer substrates in an industrial-scale PECVD reactor. Coatings.

[7] Aumaille K., Vallée C., Granier A., Goullet A., Gaboriau F., Turban G. (2000). A comparative study of oxygen/organosilicon plasmas and thin SiO_*x*_C_*y*_H_*z*_ films deposited in a helicon reactor. Thin Solid Films.

[8] Hall C.J., Murphy P.J., Griesser H.J. (2012). Etching and deposition mechanism of an alcohol plasma on polycarbonate and poly (methyl methacrylate): an adhesion promotion mechanism for plasma deposited a:SiO_*x*_C_*y*_H_*z*_ coating. Plasma Process. Polym..

[9] Zajíčková L., Buršíková V., Peřina V., Macková A., Subedi D., Janča J., Smirnov S. (2001). Plasma modification of polycarbonates. Surf. Coat. Technol..

[10] Čermák M., Kelarová Š., Jurmanová J., Kührová P., Buršíková V. (2022). The wide range optical spectrum characterization of the silicon and oxygen doped diamond like carbon inhomogeneous thin films. Diam. Relat. Mater..

[11] Zajíčková L., Buršíková V., Peřina V., Macková A., Janča J. (2003). Correlation between SiO_*x*_ content and properties of DLC: SiO_*x*_ films prepared by PECVD. Surf. Coat. Technol..

[12] Zajíčková L., Buršíková V., Kučerová Z., Franta D., Dvořák P., Šmíd R., Peřina V., Macková A. (2007). Deposition of protective coatings in rf organosilicon discharges. Plasma Sources Sci. Technol..

[13] Carneiro de Oliveira J., Airoudj A., Kunemann P., Bally-Le Gall F., Roucoules V. (2021). Mechanical properties of plasma polymer films: a review. SN Appl. Sci..

[14] Vinx N., Damman P., Leclère P., Bresson B., Fretigny C., Poleunis C., Delcorte A., Cossement D., Snyders R., Thiry D. (2021). Investigating the relationship between the mechanical properties of plasma polymer-like thin films and their glass transition temperature. Soft Matter.

[15] Mota R.P., Galvão D., Durrant S.F., De Moraes M.A.B., de Oliveira Dantas S., Cantão M. (1995). HMDSO plasma polymerization and thin film optical properties. Thin Solid Films.

[16] Cechalova B., Branecky M., Klapetek P., Cech V. (2019). Optical properties of oxidized plasma-polymerized organosilicones and their correlation with mechanical and chemical parameters. Materials.

[17] Xie X., de los Arcos T., Grundmeier G. (2022). Comparative analysis of hexamethyldisiloxane and hexamethyldisilazane plasma polymer thin films before and after plasma oxidation. Plasma Process. Polym..

[18] Poll H.U., Meichsner J., Arzt M., Friedrich M., Rochotzki R., Kreyßig E. (1993). Optical properties of plasma polymer films. Surf. Coat. Technol..

[19] Rochotzki R., Arzt M., Blaschta F., Kreyßig E., Poll H. (1993). Optical properties of plasma polymer films (hexamethyldisiloxane). Thin Solid Films.

[20] Franta D., Ohlídal I., Nečas D., Vižďa F., Caha O., Hasoň M., Pokorný P. (2011). Optical characterization of HfO_2_ thin films. Thin Solid Films.

[21] Korkmaz Ş., Pat S., Ekem N., Balbağ M.Z., Temel S. (2012). Thermal treatment effect on the optical properties of ZrO_2_ thin films deposited by thermionic vacuum arc. Vacuum.

[22] Liu M.C., Lee C.C., Kaneko M., Nakahira K., Takano Y. (2006). Microstructure-related properties at 193 nm of MgF_2_ and GdF_3_ films deposited by a resistive-heating boat. Appl. Opt..

[23] Jin J., Jin C., Li C., Deng W., Yao S. (2015). Influence of substrate temperatures on the properties of GdF_3_ thin films with quarter-wave thickness in the ultraviolet region. Appl. Opt..

[24] Azzam R.M.A., Elshazly-Zaghloul M., Bashara N.M. (1975). Combined reflection and transmission thin-film ellipsometry: a unified linear analysis. Appl. Opt..

[25] Pei L., Jiaqi Z., Yuankun Z., Jiecai H. (2013). Preparation and optical properties of sputtered-deposition yttrium fluoride film. Nucl. Instrum. Methods Phys. Res. B.

[26] Vedam K., McMarr P.J., Narayan J. (1985). Nondestructive depth profiling by spectroscopic ellipsometry. Appl. Phys. Lett..

[27] Kildemo M. (1998). Real-time monitoring and growth control of Si-gradient-index structures by multiwavelength ellipsometry. Appl. Opt..

[28] Carniglia C.K. (1990). Ellipsometric calculations for nonabsorbing thin films with linear refractive-index gradients. J. Opt. Soc. Am. A.

[29] Jacobsson R., Wolf E. (1966).

[30] Sheldon B., Haggerty J.S., Emslie A.G. (1982). Exact computation of the reflectance of a surface layer of arbitrary refractive-index profile and an approximate solution of the inverse problem. J. Opt. Soc. Am..

[31] Franta D., Vohánka J., Dvořák J., Franta P., Ohlídal I., Klapetek P., Březina J., Škoda D. (2023). Optical characterization of gadolinium fluoride films using universal dispersion model. Coatings.

[32] Ohlídal I., Vohánka J., Buršíková V., Ženíšek J., Vašina P., Čermák M., Franta D. (2019). Optical characterization of inhomogeneous thin films containing transition layers using the combined method of spectroscopic ellipsometry and spectroscopic reflectometry based on multiple-beam interference model. J. Vac. Sci. Technol. B.

[33] Franta D., Ohlídal I., Klapetek P., Montaigne Ramil A., Bonanni A., Stifter D., Sitter H. (2002). Influence of overlayers on determination of the optical constants of ZnSe thin films. J. Appl. Phys..

[34] Ohlídal M., Ohlídal I., Klapetek P., Nečas D., Majumdar A. (2011). Measurement of the thickness distribution and optical constants of non-uniform thin films. Meas. Sci. Technol..

[35] Vohánka J., Franta D., Čermák M., Homola V., Buršíková V., Ohlídal I. (2020). Ellipsometric characterization of highly non-uniform thin films with the shape of thickness non-uniformity modeled by polynomials. Opt. Express.

[36] Richter U. (1998). Application of the degree of polarization to film thickness gradients. Thin Solid Films.

[37] Pisarkiewicz T. (1994). Reflection spectrum for a thin film with non-uniform thickness. J. Phys. D, Appl. Phys..

[38] Nagata K., Nishiwaki J. (1967). Reflection of light from filmed rough surface: determination of film thickness and rms roughness. Jpn. J. Appl. Phys..

[39] Bauer J. (1977). Optical properties, band gap, and surface roughness of Si_3_N_4_. Phys. Status Solidi A.

[40] Ohlídal I., Vohánka J., Buršíková V., Dvořák J., Klapetek P., Kaur N.J. (2022). Optical characterization of inhomogeneous thin films with randomly rough boundaries exhibiting wide intervals of spatial frequencies. Opt. Eng..

[41] Vohánka J., Ohlídal I., Buršíková V., Klapetek P., Kaur N.J. (2022). Optical characterization of inhomogeneous thin films with randomly rough boundaries. Opt. Express.

[42] Martinu L., Poitras D. (2000). Plasma deposition of optical films and coatings: a review. J. Vac. Sci. Technol., A, Vac. Surf. Films.

[43] Franta D., Ohlídal I. (2000). Analysis of thin films by optical multi-sample methods. Acta Phys. Slovaca.

[44] Fujiwara H. (2007).

[45] Germer T., Zwinkels J.C., Tsai B.K. (2014).

[46] Franta D., Nečas D., Ohlídal I. (2015). Universal dispersion model for characterization of optical thin films over wide spectral range: application to hafnia. Appl. Opt..

[47] Franta D., Vohánka J., Čermák M., Stenzel O., Ohlídal M. (2018). Optical Characterization of Thin Solid Films.

[48] Peter S., Graupner K., Grambole D., Richter F. (2007). Comparative experimental analysis of the aC: H deposition processes using CH4 and C2H2 as precursors. J. Appl. Phys..

[49] Campi D., Coriasso C. (1988). Prediction of optical properties of amorphous tetrahedrally bounded materials. J. Appl. Phys..

[50] Franta D., Mureşan M.G. (2021). Wide spectral range optical characterization of yttrium aluminum garnet (YAG) single crystal by the universal dispersion model. Opt. Mater. Express.

[51] Dvořák J., Vohánka J., Buršíková V., Franta D., Ohlídal I. (2023). Optical characterization of inhomogeneous thin films deposited onto non-absorbing substrates. Coatings.

[52] Ohlídal I., Vohánka J., Buršíková V., Franta D., Čermák M. (2020). Spectroscopic ellipsometry of inhomogeneous thin films exhibiting thickness non-uniformity and transition layers. Opt. Express.

[53] Ohlídal I., Vohánka J., Čermák M. (2021). Optics of inhomogeneous thin films with defects: application to optical characterization. Coatings.

[54] Ohlídal I., Vohánka J., Buršíková V., Šulc V., Šustek Š., Ohlídal M. (2020). Ellipsometric characterization of inhomogeneous thin films with complicated thickness non-uniformity: application to inhomogeneous polymer-like thin films. Opt. Express.

[55] Franta D., Nečas D., Vohánka J. Software for optical characterization newAD2. http://newad.physics.muni.cz/.

[56] Ohlídal I., Vohánka J., Mistrík J., Čermák M., Vižďa F., Franta D. (2019). Approximations of reflection and transmission coefficients of inhomogeneous thin films based on multiple-beam interference model. Thin Solid Films.

[57] Tyczkowski J. (2004). Plasma Polymer Films.

[58] Catherine Y., Turban G. (1979). Reactive plasma deposited SixCyHz films. Thin Solid Films.

[59] Borvon G., Goullet A., Mellhaoui X., Charrouf N., Granier A. (2002). Electrical properties of low-dielectric-constant films prepared by PECVD in O2/CH4/HMDSO. Mater. Sci. Semicond. Process..

[60] Brzobohatý O., Buršíková V., Trunec D. (2004). Mirror effect in PECVD reactor and its explanation via MC-PIC computer simulation. Czechoslov. J. Phys..

[61] Brzobohatý O., Buršíková V., Nečas D., Valtr M., Trunec D. (2008). Influence of substrate material on plasma in deposition/sputtering reactor: experiment and computer simulation. J. Phys. D, Appl. Phys..

[62] Taccogna F., Longo S., Capitelli M. (2004). Plasma-surface interaction model with secondary electron emission effects. Phys. Plasmas.

[63] Taccogna F., Longo S., Capitelli M. (2004). Effects of secondary electron emission from a floating surface on the plasma sheath. Vacuum.

[64] Jolivet L., Roussel J.F. (2002). Numerical modeling of plasma sheath phenomena in the presence of secondary electron emission. IEEE Trans. Plasma Sci..

